# HIV and Malnutrition: Effects on Immune System

**DOI:** 10.1155/2012/784740

**Published:** 2012-01-02

**Authors:** Shalini Duggal, Tulsi Das Chugh, Ashish Kumar Duggal

**Affiliations:** ^1^Department of Microbiology, Dr. Baba Saheb Ambedkar Hospital, Rohini, New Delhi 110085, India; ^2^Department of Microbiology, BLK Superspeciality Hospital, Pusa Road, New Delhi 110005, India; ^3^Department of Medicine, Dr. Ram Manohar Lohia Hospital & PGIMER, Baba Kharag Singh Marg, New Delhi 110001, India

## Abstract

HIV or human immunodeficiency virus infection has assumed worldwide proportions and importance in just a span of 25 years. Continuous research is being done in many parts of the world regarding its treatment and vaccine development, and a lot of money has flown into this. However, fully understanding the mechanisms of immune depletion has still not been possible. The focus has also been on improving the quality of life of people living with HIV/AIDS through education, counselling, and nutritional support. Malnutrition further reduces the capacity of the body to fight this infection by compromising various immune parameters. Knowledge of essential components of nutrition and incorporating them in the management goes a long way in improving quality of life and better survival in HIV-infected patients.

## 1. Introduction

HIV accounts for significant immunosuppression in an infected individual. If the corroboratory indices of good health are satisfactory, the suppression of immune defences can be mitigated. One such index is nutrition. HIV, immune expression, and nutrition interactions are complex and related to each other. Malnutrition adds fuel to the fire by accelerating the progress of HIV infection to AIDS. HIV/AIDS is associated with biological and social factors that affect the individual's ability to consume, utilize, and acquire food. Once there is an infection with HIV, the patient's nutritional status declines further leading to immune depletion and HIV progression.

## 2. Review of the Literature

Acquired immune deficiency syndrome, or AIDS, is a disease caused by a retrovirus, the human immunodeficiency virus (HIV), which attacks and impairs body's natural defence system against disease and infection. Malnutrition is defined as “the cellular imbalance between supply of nutrients and energy and the body's demand for them to ensure growth, maintenance, and specific functions” [1]. Nutrition and HIV are strongly related and complement each other. HIV causes immune impairment leading to malnutrition which leads to further immune deficiency, and contributes to rapid progression of HIV infection to AIDS. A malnourished person after acquiring HIV is likely to progress faster to AIDS, because his body is weak to fight infection whereas a well-nourished person can fight the illness better. It has been proved that good nutrition increases resistance to infection and disease, improves energy, and thus makes a person stronger and more productive. Wasting syndrome is defined by loss of more than 10% of the usual body weight with a lack of other detectable cause of wasting other than the HIV infection itself [2]. Nutritional improvement measures must be initiated before a patient reaches this stage. 

### 2.1. Malnutrition in HIV

One of the factors responsible for malnutrition in an HIV-infected person is reduced appetite, which could be due to difficulty in ingesting food as a result of infections like oral thrush or oesophagitis caused by *Candida,* a common opportunistic infection in HIV-infected people and fever, side effects of medicines, or depression. Poor absorption of nutrients may be due to accompanying diarrhea which may be because of bacterial infections like *Salmonella* or *Mycobacterium avium intercellular*; viral like CMV or parasitic infections like *Giardia, C. parvum*, and *E. Bieneusi;* due to nausea/vomiting as a side effect of medications used to treat HIV or opportunistic infections. 30–50% of HIV patients in developed and nearly 90% in developing countries complain of diarrhoea and malabsorption [3]. Gastrointestinal tract is the largest lymphoid organ in the body and is directly affected by HIV infection. HIV causes damage to the intestinal cells by causing villus flattening and decreased D-xylose absorption. This leads to carbohydrate and fat malabsorption thereby affecting fat soluble vitamins like vitamins A and E, which are important for proper functioning of immune system. Whereas larger amounts of nutrients are required during fever and infections that accompany an HIV infection, they are utilised poorly by the body. This leads to loss of weight and lean muscle tissue, further causing damage to the immune system. Lack of iron in the diet and infections such as malaria and hookworm lead to anaemia. Anaemia causes lethargy, further reduces food intake and nutrient absorption, and also causes disruption of metabolism, chronic infections, muscle wasting, or loss in lean body tissue [4]. AIDS-related dementia or neuropsychiatric impairment may make the patients unable to care for themselves, forget to eat, or unable to prepare balanced meals. Even in households with HIV-infected members, nutritional impacts can be seen if the infected adult becomes too sick to work and provide food for themselves and their families [5, 6]. Dietary intake also varies inversely with level of virus, suggesting that viral replication directly or indirectly suppresses appetite [7]. Malnutrition is frequent and is considered a marker for poor prognosis among HIV-infected subjects [8].

### 2.2. Immunology of HIV Infection

Both CD4+ and CD8+ T cells are important in controlling HIV infection. HIV infection stimulates production of cytokines such as TNF*α*, IL-6, IL-10, and IFN*γ* and a pool of activated target cells in the lymphoid tissue which paradoxically help in establishing and propagating HIV infection. Rosenberg et al. [9] observed that HIV-specific CD4+ T-cell responses were of high magnitude in individuals who were HIV infected but not showing progression over long periods (long-term nonprogressors). Also, in acute viral infections such responses could be seen but they were generally not present in patients with chronic progressive infections. In a small number of individuals who began treatment shortly after acute HIV infection, HIV-specific CD4+ T-cell responses were preserved. In addition, these CD4+ T-cell responses seem to be important in controlling viral replication after subsequent discontinuation of antiretroviral therapy. However, it has also been found that when discontinuation of antiretroviral therapy leads to loss of virologic control, HIV-specific CD4+ T cells are preferentially infected and depleted compared with the CD4+ T cells of other antigen specificities. Antiviral immunity involves both the arms of the immune system. The protective component of cell-mediated immunity involves the cytotoxic CD8 T-lymphocytes. Schmitz and colleagues had demonstrated the effects of CD8 T lymphocytes in monkeys experimentally infected with simian immunodeficiency virus (SIV). They observed that prior to the depletion of CD8+ T cells, SIV replication was well controlled but after their depletion, control of viral replication was lost. In some of these monkeys, when the CD8+ T cells regenerated, the control of viral replication was regained [10]. Humoral immunity to HIV is expressed by neutralising antibodies. Anti-HIV antibodies are able to bind cell-free virus and potentially prevent established infection in the challenged host. Neutralising antibodies attaching to CD4 binding site of HIV have been identified which appear to prevent the virus from attaching to and infecting T cells. These are natural human antibodies—named VRC01, VRC02, and VRC03 which can neutralize over 90% of circulating HIV-1 isolates [11]. Though HIV-specific humoral immune responses can be detected during primary infection, they mostly comprise low-avidity env specific IgG antibodies with little or no neutralising activity [12]. Significant neutralising titers are believed to take place after chronicity has set in. HIV evolves various strategies to establish chronicity in human body. These include viral latency, inhibition of antigen processing or presentation, mutations in viral epitopes, and rapid clonal exhaustion/deletion of the initially expanded virus-specific CD8+ CTL clones [13]. Initial CTL responses cause downregulation of viremia and prevent disease progression, but later it induces the selection of virus mutants capable of escaping the immune response [14]. HIV virions concentrate on the surface of follicular dendritic cells in the germinal centres of lymphoid organs from where they are shed intermittently to establish a steady chronic state of infection of CD4+ T cells, and to a chronic inflammatory reaction that ultimately results in the destruction of lymphoid tissue [14]. Immune activation in HIV is supported by an experiment by Pandrea et al. where induction of immune activation was demonstrated in nonpathogenic SIV infection by an increase in viral replication and CD4+ T-cell depletion in gut associated lymphoid tissue [15]. Immune activation is attributed to bystander activation in response to viral products including gene products, immune response to HIV, translocated microbial products, new viral target proteins, epithelial or immune cells apoptosis, and/or self-antigens [16]. High T-cell turnover in chronic HIV infection is attributed to overlapping and nonsynchronized bursts of proliferation, differentiation, and death in response to T-cell receptor- (TCR-) mediated stimulation and inflammation [16, 17]. Antiretroviral therapy (ART) results in a marked reduction of T-cell activation and apoptosis and helps to decrease naive T-cell consumption and restore their numbers [18]. Chronic HIV infection also causes immunological or direct virotoxic effects on gastrointestinal tract which shows blunted villi, crypt hyperplasia, and damaged epithelial barrier with increased permeability and malabsorption of bile acid and vitamin B12, microbial translocation, and enterocyte apoptosis. There is a decrease of luminal defensins and massive CD4 T-cell depletion but high concentration of infected CD4 T cells [19].

### 2.3. Immunology of Malnutrition

Malnutrition is considered to be the most common cause of immunodeficiency worldwide [20]. Malnutrition, immune system, and infectious diseases are interlocked in a complex negative cascade [1]. Malnutrition elicits dysfunctions in the immune system and promotes increased vulnerability of the host to infections [21]. These immune dysfunctions are referred to as nutritional-acquired immune deficiency syndrome (NAIDS). Every type of immunological deficiency induced by malnutrition can be included under the NAIDS umbrella.

#### 2.3.1. PEM

Protein-energy malnutrition (PEM), now known as protein-energy undernutrition, is an energy deficit due to chronic deficiency of all macronutrients [22]. In children, PEM causes widespread atrophy of lymphoid tissues, particularly T-lymphocyte areas. The thymus involutes causing a reduction in the thymus-derived lymphocyte growth and maturation factors, arrest of lymphocyte development, reduced numbers of circulating mature CD4 helper cells, and impairment of antibody production to T-dependent antigens. Imbalance in Th1-Th2 activation occurs depending on nature of stimuli and altered regulatory pathways, including responses mediated by the nuclear factor-kB (NF-kB) [23], a major transcription factor involved in the development of innate and adaptive immunity. Hence the patient's ability to ward off infections and show recovery is compromised. However, CD8 suppressor cells are relatively preserved. The lymphocytes not only get reduced in blood, but also impaired show T-lymphocyte mitogenesis and diminished activity in response to mitogens [24]. According to Chandra [25], in children with PEM, there is a decrease or reversal of the T-helper-suppressor cell ratio and total numbers of T-lymphocytes decrease due to reduced numbers of these T-cell subpopulations. In malnourished children, changes such as dermal anergy, loss of delayed dermal hypersensitivity (DDH) reactions, and loss of the ability of killer lymphocytes to recognize and destroy foreign tissues were noted [20]. Necropsy studies on malnourished patients have also shown profound depletion of the thymolymphatic system and severe depression of cell-mediated immunity. Chronic thymic atrophy with peripheral lymphoid tissue wasting along with depletion of paracortical cells and loss of germinal centres was noted. This was suggested to have led to various types of infections from which these patients actually died [26].

 B-lymphocyte numbers and functions generally appear to be maintained though immunoglobulin concentrations get reduced including secretory IgA (sIgA), which is responsible for mucosal immunity. This may be due to increased bacterial adherence to nasopharyngeal and buccal epithelial cells or altered expression of membrane glycoprotein receptors [27]. It has been speculated that the existing antibody production is conserved or even increased during generalized malnutrition but new primary antibody responses to T-cell-dependent antigens and antibody affinity are impaired [20]. The failure of antibody formation is reversed within a few days of protein therapy as amino acids become available for the synthesis of immune proteins [28]. It also reduces complement formation, and interferon and lower interleukin 2 receptors [26]. In patients with severe generalized malnutrition, functional status of the immune system should be assessed by simply looking at the tonsils in young children. In adequately nourished children they are usually huge but are virtually undetectable in children with severe PEM. This would indicate atrophy in the child's thymus, spleen, and lymph nodes, and severely compromised cell-mediated immunity [24].

Deficiencies of other nutrients also adversely affect the immune mechanisms. Deficiencies of essential amino acids can depress the synthesis of proteins responsible for production of cytokines released by lymphocytes, macrophages, and other body cells, complement proteins, kinins, clotting factors, and tissue enzymes activated during acute phase responses [24]. Arginine deficiency diminishes the production of nitric oxide, and hence, the antioxidants, allowing damaging effects of free oxygen radicals [24]. Arginine has also been shown to enhance phagocytes of alveolar macrophages, depress T suppressor cells, and stimulate T helper cells [29]. The “nonessential” amino acid glutamine is necessary for lymphocytes and other rapidly growing cells.

#### 2.3.2. Essential Fatty Acids

Particularly the omega-3 fatty acids, serve as the key precursors for the production of eicosanoids like prostaglandins, prostacyclins, thromboxanes, and leukotrines that play a variety of host defensive roles. Thus their deficiency in the diet can impair cytokine synthesis [30].

#### 2.3.3. Vitamins

Vitamin A has an important role in nucleic acid synthesis, and its deficiency is also characterized by lymphoid tissue atrophy, depressed cellular immunity, impaired IgG responses to protein antigens, and pathologic alterations of mucosal surfaces. Experimental animals with vitamin A deficiency have decreased thymus and spleen sizes, reduced natural killer cell, macrophage and lymphocyte activity, lower production of interferon, and weak response to stimulation by mitogens [31]. B-group vitamins like thiamin, riboflavin, pantothenic acid, biotin, folic acid, and cobalamin can influence humoral immunity by diminishing antibody production. Pyridoxine deficiency has also been associated with reduced cell-mediated immunity. Folic acid and vitamin B-12 are essential to cellular replication. Experimental deficiencies of these vitamins were shown to interfere with both replication of stimulated leukocytes and antibody formation. In anemia due to folic acid deficiency, cell-mediated immunity is depressed [32]. In vitamin C deficiency, phagocytic cells cannot produce tubulin, therefore, with impaired chemotaxis, microorganisms cannot be engulfed and destroyed [33]. Vitamin D acts as an immunoregulatory and a lymphocyte differentiation hormone [34]. In vitamin E deficiency, leukocyte especially lymphocyte killing power gets reduced. In animals it was shown to interfere with antibody formation, plaque-forming cells, and other aspects of cell-mediated immunity. At higher than recommended levels, it has been shown to enhance immune response and resistance to disease [35].

#### 2.3.4. Minerals

Zinc is also the fundamental component of thymic hormones and shares a similar role as vitamin A in nucleic acid synthesis. Zinc deficiency influences both lymphocyte and phagocyte cell functions and affects more than 100 metalloenzymes that are zinc dependent [36]. During infections, reticuloendothelial cells sequester iron from the blood and phagocytes release lactoferrin with a higher iron binding capacity than bacterial siderophores. The net effect is to deprive the infectious agent of iron for its replication and inhibit the spread of infection [34]. Iron deficiency results in impaired phagocytic killing, less response to lymphocyte stimulation, fewer natural killer cells, and reduced interferon production [37]. Selenium serves as an antioxidant and contributes to antibody responses and cytotoxicity of natural killer cells [38]. In children with HIV infection, selenium concentration in plasma appeared to correlate with their immune functions [39]. Similar changes were also seen in patients with copper deficiency [40]. Copper concentrations often increase during infection as a result of stimulation of the hepatic production of ceruloplasmin. Conversely, plasma zinc concentration often declines due to internal redistribution to the liver. Antimicrobial systems in the neutrophils are affected by malnutrition. These include both oxygen-dependent systems responsible for the respiratory burst, and oxygen-independent systems, such as lactoferrin, lysozymes, hydrolase, and proteases [34].

### 2.4. Cytokine Abnormalities in HIV and/or Malnutrition

Cytokines are substances that play an important role in coordinating inflammatory response of the body to various external and internal stimuli. They may be proinflammatory, which are essential to initiate defence against various pathogens, and anti-inflammatory, which downregulate the inflammatory process by suppressing production of the proinflammatory cytokines and balance the inflammatory response. Excess production of both are counterproductive. The proinflammatory cytokines include IL-1*β*, IL-6, IL-8, TNF-*α*, and IL-2, and the anti-inflammatory cytokines include IL-1 receptor antagonist, IL-4, IL-10, and IL-13 [41]. PEM diminishes immunoglobulin (IgA, IgM, and IgG) concentrations and cytokine production [34]. Severe malnutrition alters the ability of T lymphocytes to respond appropriately to IL-1 rather than simply affecting synthesis of this monokine [42]. During catabolic states, interleukin 1 is released by leukocytes which causes endocrine changes that lead to amino-acid mobilization, primarily from skeletal muscle. These amino acids are used for gluconeogenesis in the liver, and the nitrogen released is excreted in urine [43]. Thus, a continual conversion of alanine carbon to glucose carbon occurs with acute infection. Bell et al. [44] observed that the immunosuppressive PGE2 production was enhanced in malnutrition. In malnourished Africans without overt infections, increased circulating levels of inflammatory mediators (e.g., interleukin 6 (IL-6), the soluble receptors of tumor necrosis factor (sTNFR-p55 and sTNFR-p75), etc.) as well as C-reactive protein, were seen compared to healthy controls [45]. In HIV infection, both CD4+ and CD8+ T cells secrete interferon-*γ* (IFN-*γ*) in response to antigen-specific stimulation. Another cytokine, tumor necrosis factor has been suggested as a potential etiologic factor in HIV wasting syndrome as it has been incriminated as an appetite inhibitor [46].

### 2.5. Combined Effect of HIV and Malnutrition on the Immune System

Malnutrition and HIV form a vicious cycle and ultimately aim at reducing the immunity of the patient. In both malnutrition and HIV there is reduced CD4 and CD8 T-lymphocyte numbers [47], delayed cutaneous sensitivity, reduced bacteriocidal properties [24], and impaired serological response after immunizations. Some of the immunological parameters concerning these two entities have been listed in Table 1. According to a study, approximately 30–60% of asymptomatic children infected with HIV malabsorb carbohydrates, 30% malabsorb fat, and 32% malabsorb proteins [48, 49]. Whereas micronutrient deficiencies may affect replication of the invading virus, they also induce several metabolic alterations in the body. This includes changes in whole-body protein turnover, increased urinary nitrogen loss, and elevated hepatic protein synthesis as well as increased skeletal muscle breakdown providing for proliferation of neutrophils, lymphocytes, and fibroblasts, and for synthesis of immunoglobulins and hepatic acute phase proteins, manifesting clinically as fever. It also includes hypertriglyceridemia, elevated hepatic de novo fatty acid synthesis, decreased peripheral lipoprotein lipase activity, hyperglycemia, insulin resistance, and increased gluconeogenesis. Serum concentrations of iron and zinc fall dramatically due to redistribution within the body, with accumulation in the liver [50]. Glutathione, which is the principle intracellular antioxidant, was reported to be reduced in children with HIV infection, especially those showing growth failure [51].

During infections, reactive oxygen molecules and pro-oxidant cytokines are released from activated phagocytes [56] leading to increased consumption of vitamins like vitamin E and C, and *β*-carotene which serve as antioxidants and minerals like zinc, copper, manganese, and selenium, which serve as components of antioxidant enzymes [57]. Deficiencies of antioxidants cause increased oxidative stress which leads to apoptosis of T cells and indirectly compromise cell-mediated immunity and may stimulate HIV replication. In cell cultures, HIV replication was shown to be inhibited by various antioxidants but stimulated by reactive oxygen radicals via activation of nuclear transcription factor cell gene [58]. This oxidative burst may also increase viral load of blood and body fluids, such as seminal fluid and cervicovaginal secretions, and thus increase infectivity. Maternal micronutrient deficiencies may also increase viral load in blood, cervicovaginal secretions, and breast milk, and hence aid in utero, intrapartum, and postnatal mother-to-child HIV transmission, respectively, and affect immune functions and susceptibility of the unborn or young breast-fed child. HIV infection in nutritionally deprived individuals intensifies the nutritional deficits and further enhances cellular oxidative stress. This affects the functions of transcription factors as NF-kB and contributes to HIV replication and progression. Although HIV attacks only a limited variety of T-lymphocyte subspecies, AIDS-induced malnutrition can lead to the secondary development of NAIDS through the action of proinflammatory cytokines. Also, malnutrition could hasten the development of AIDS in an HIV-infected person [24].

Specific micronutrient deficiencies may also favour the host and supplementation favour the virus. For example, HIV replication was enhanced in monocytes cultured with retinoid [59]. Similarly, HIV nucleocapsid protein binds zinc and forms zinc finger structures. This might imply that a high zinc intake increases the replication of HIV [60]. The role of iron in HIV infection is more complex, since iron is important for optimal immune function, and is also a pro-oxidant and may promote replication, as has been shown following a U-shaped curve in laboratory studies [61]. Though antioxidants inhibit HIV replication, they may actually promote opportunistic infections by preventing the oxidative burst which is considered important for the bactericidal properties of phagocytes [56]. So, balanced nutrition and dietary consultation with experts helps in balancing immune effects, malnutrition, and HIV infection. FAO [62] had stated “Food is not a magic bullet. It won't stop people from dying of AIDS but it can help them live longer, more comfortable and productive lives.” Evidence-based nutrition interventions should be part of all national HIV care and treatment programmes. Routine assessment should be made of diet and nutritional status (weight and weight change, height, body mass index or midupper arm circumference, and symptoms and diet) for people living with HIV [63].

Baum et al. [64] had concluded that “intake of nutrients at levels recommended for the general population does not appear to be adequate for HIV-1-infected patients'.” An active non-HIV-infected adult requires approximately 2070 kcal/day including about 57 grams/day of protein. An HIV-infected adult requires 10 to 15 percent more energy per day and approximately 50 to 100 percent more protein [65, 66]. Diet given to such patients should be rich in carbohydrates, proteins, vitamins, and minerals. A dietician should be involved in guiding the patients or their relatives to prepare nutritious foods. In developing countries, micronutrient supplementation to high-risk populations can be provided via the primary health care system.

Since the realization of HIV as a potential disaster for the immune system, several advancements in its treatment, diagnosis, and supportive regimens have been made, still many deaths in AIDS are being attributed to malnutrition and its poor management. Biochemical evaluation of the nutritional status must be done in AIDS patients by testing blood haemoglobin and haematocrit and serum levels of cholesterol, total protein, albumin, and transferrin. Nutritional counselling and support could delay or even prevent the development of NAIDS and could improve both the quality and length of their lives. Therefore, early and intensive dietary interventions should be a fundamental part of the case management of HIV-infected individuals at the level of ART centre itself.

## Figures and Tables

**Table 1:** Comparison of effects of HIV infection and malnutrition on various parameters of the immune system [20, 24, 47, 52–55].

Immunological parameter	Effect of HIV infection	Effect of malnutrition	Nutrient deficiency
Total lymphocytes	Decreased	Decreased	PEM
T lymphocytes	Decreased	Decreased	PEM
CD4 T lymphocytes	Decreased	Decreased	PEM
CD8 T lymphocytes	Transient increase, then decrease	Relatively maintained	—
CD4: CD8 T-cell ratio	Inverted	Reversed	PEM
Lymphocyte responsiveness to mitogens/antigens	Reduced	Reduced	PEM, vitamin A, E, zinc, iron
Cell-mediated immunity	Compromised	Compromised	PEM, essential amino acids (pyridoxine)
B lymphocytes	Polyclonal activation	Generally maintained	—
Immunoglobulin levels	Increased (IgA, IgG)	Reduced (IgA, IgG, IgM)	PEM, amino acids, vitamin B complex
Secretory IgA (sIgA)	Increased	Decreased	PEM
B-cell activity	Reduced	Reduced	PEM,
Primary antibody responses	Reduced	Reduced	PEM
Antibody affinity	Decreases with increase in HIV progression	Reduced	PEM
NK cell activity	Increased	Reduced	Vitamin A, C, zinc, iron, selenium
Serum complement	Increased	Reduced	PEM, essential amino acids
Serum *β*-2 microglobulin	Increased, marker of HIV progression	Increased	PEM
IFN-*γ*	Increased	Reduced	Amino acids, essential fatty acids, iron
TNF-*α*, IL-6	Increased	Increased	PEM
Anti-inflammatory Cytokines (IL-4)	Reduced	Increased	PEM
Soluble IL-2 receptors	Increased	Reduced	PEM
Antioxidants	Reduced	Reduced	Essential amino acids (arginine), selenium, zinc, manganese, copper, vitamins A, C, E
C-reactive protein	Increased, marker of HIV disease progression	Increased	PEM
